# Birth and Rapid Subcellular Adaptation of a Hominoid-Specific CDC14 Protein

**DOI:** 10.1371/journal.pbio.0060140

**Published:** 2008-06-10

**Authors:** Lia Rosso, Ana Claudia Marques, Manuela Weier, Nelle Lambert, Marie-Alexandra Lambot, Pierre Vanderhaeghen, Henrik Kaessmann

**Affiliations:** 1 Center for Integrative Genomics, University of Lausanne, Lausanne, Switzerland; 2 Institut de Recherches en Biologie Humaine et Moleculaire (IRIBHM), University of Brussels, Brussels, Belgium; University of Chicago, United States of America

## Abstract

Gene duplication was prevalent during hominoid evolution, yet little is known about the functional fate of new ape gene copies. We characterized the *CDC14B* cell cycle gene and the functional evolution of its hominoid-specific daughter gene, *CDC14Bretro*. We found that *CDC14B* encodes four different splice isoforms that show different subcellular localizations (nucleus or microtubule-associated) and functional properties. A microtubular *CDC14B* variant spawned *CDC14Bretro* through retroposition in the hominoid ancestor 18–25 million years ago (Mya). *CDC14Bretro* evolved brain-/testis-specific expression after the duplication event and experienced a short period of intense positive selection in the African ape ancestor 7–12 Mya. Using resurrected ancestral protein variants, we demonstrate that by virtue of amino acid substitutions in distinct protein regions during this time, the subcellular localization of CDC14Bretro progressively shifted from the association with microtubules (stabilizing them) to an association with the endoplasmic reticulum. CDC14Bretro evolution represents a paradigm example of rapid, selectively driven subcellular relocalization, thus revealing a novel mode for the emergence of new gene function.

## Author Summary

Many new gene copies emerged by gene duplication in human and ape (hominoid) ancestors. However, little is known with respect to their functional evolution. We used a combination of evolutionary analyses and cell biology experiments to unveil the adaptive evolution of the hominoid-specific *CDC14Bretro* gene, which arose as a reverse-transcribed copy of a messenger RNA of its “parent,” the *CDC14B* cell cycle gene. We first show that the *CDC14B* parental gene encodes different splice isoforms that differ with respect to their subcellular localization and functional properties. One of these isoforms, which is associated with microtubules throughout the cell cycle (stabilizing them and potentially contributing to their organization), spawned *CDC14Bretro* in the common ape ancestor 18–25 million years ago. In the African ape (human/chimp/gorilla) ancestor, around 7–12 million years ago, intense positive selection then led to rapid relocalization of the CDC14Bretro protein from microtubules to a new cellular location—the endoplasmic reticulum. This radical subcellular shift likely reflects the evolution of a new function of CDC14Bretro in the African ape lineage. In contrast, CDC14Bretro retained the ancestral (parental) localization and function in Asian apes (orangutans and gibbons). Our study not only adds to the rare known cases of ape-specific genes for which selectively driven substitutions could be tied to functional protein change and adaptation, but, more generally, reveals a novel mode that may underlie the emergence of new gene function: rapid, selectively driven subcellular adaptation.

## Abstract 3

Evolutionary and experimental analyses of the hominoid CDC14 retrogene uncover a novel mode for the emergence of new gene function: selectively driven subcellular adaptation of the encoded protein.

## Introduction

Gene duplication has been important for the evolution of phenotypes specific to species and evolutionary lineages by providing the genetic raw material for the emergence of genes with new or altered functions [1]. Duplicate gene copies are commonly generated through duplication of gene-containing chromosomal segments (segmental duplication; [2]), or by reverse-transcription of mRNAs from parental source genes (termed retroduplication or retroposition), generating intronless gene copies (retrocopies) of the parent [3,4]. However, although both of these mechanisms were shown to have generated a significant number of gene copies during recent primate evolution on the lineage leading to humans [5–7], little is known with respect to their functional evolution and impact on phenotypes typical to humans and their close evolutionary primate relatives.

Indeed, only a relatively small number of functionally preserved segmentally duplicated genes or functional retrocopies (retrogenes) that emerged in hominoids (humans and apes) have been pinpointed [6,8–12]. Although some of these genes revealed striking signatures of positive selection [10], selectively driven substitutions could only rarely be tied to functional protein change and adaptation [8]. Even in primates in general, few cases of new duplicate genes exist where amino acid substitutions resulting from positive selection were experimentally demonstrated to lead to functional adaptation of the encoded protein [13,14].

**Figure 1:**
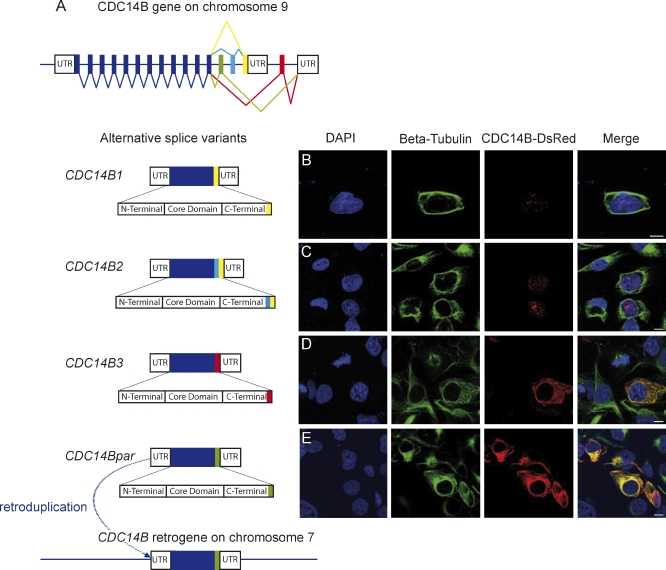
Splice Variants of the *CDC14B* Parental Gene and Subcellular Localization of Encoded Proteins (A) Exon and protein structures of *CDC14B* splice variants. Color code of coding exons (exons not drawn to scale): exons shared by all variants are colored in dark blue; exons colored in yellow, light blue, green, and red are specific to transcripts *CDC14B1*, *CDC14B2*, *CDC14B3*, and *CDC14Bpar*, respectively, as indicated by the schematic representation of splicing patterns. The basic protein structure and sequence encoded by the different splice variants is indicated (only the protein sequence that differs between the splice variants is color-coded to indicate the relationship with the alternative splicing patterns of the coding exons). The retroduplication event of the *CDC14Bpar* transcript that gave rise to the *CDC14B* retrogene (*CDC14Bretro*) is illustrated. (B–E) COS7-cells (from African green monkeys—no endogenous *CDC14Bretro* gene) transfected with *CDC14B*-DsRed fusion constructs (i.e., CDC14B signals in red) are shown. Microtubules were stained using an anti-beta tubulin antibody (green) and the nucleus was stained using DAPI (blue). Merged images: CDC14B1 (nucleoli) and −2 (nuclear speckles) localize to the nucleus (violet) and CDC14B3 and CDC14Bpar co-localize with microtubules (yellow). We obtained very similar results when using HeLa or LN229 (glioblastoma) cell lines (both carrying an endogenous *CDC14Bretro* gene, Figure S1). Thus, there appear to be no cell line–specific effects with respect to the observed subcellular localization patterns. Scale bars (white) = 10 μm.

In a previous survey [6], we identified an interesting retrogene, termed *CDC14Bretro* (denoted *CDC14B2* in [6]), which originated by retroduplication (from Chromosome 9 to 7) from its parent, *CDC14B*, in the hominoid ancestor around 18–25 million years ago (Mya) [15] (Figure 1A). *CDC14B* and its paralog *CDC14A* were recently shown to represent the mammalian counterparts of the single, dual-specificity phosphatase gene *CDC14* from yeast, a key regulator of late mitotic events, which is particularly important for mitotic exit [16]. Although both paralogs can rescue *CDC14* deletion mutants of Schizosaccharomyces pombe (fission yeast), *CDC14B* was shown to be the functional ortholog of *CDC14* from budding yeast [17]. Prompted by the potential functional implications of an additional *CDC14* family member for the phenotypic evolution of hominoids, we set out to characterize the functional evolution of the *CDC14B* daughter gene, *CDC14Bretro*.

## Results and Discussion

### The Human *CDC14B* Parental Gene Encodes Four Different Alternative Splice Variants

To understand the origin of *CDC14Bretro*, we first characterized the transcript that gave rise to *CDC14Bretro*, because two *CDC14B* splice variants have been analyzed in previous studies [17–19] (we have termed them *CDC14B1* and *CDC14B2*, Figure 1A). To this end, we aligned the *CDC14Bretro* sequence to the genomic locus of its parental gene on Chromosome 9. This procedure revealed a previously undescribed exon structure, indicating a transcript (the “parent”—termed *CDC14Bpar*—that gave rise to *CDC14Bretro*) with a previously unknown 3′ splicing pattern (Figure 1A). A fourth 3′-end splice variant (*CDC14B3*) is annotated (Ensembl database [20]) and supported by expressed sequence tags (Figure 1A). Thus, the human *CDC14B* gene encodes at least four different alternative splice variants.

### Expression of the *CDC14B* Splice Variants and the *CDC14Bretro* Daughter Gene

**Figure 2:**
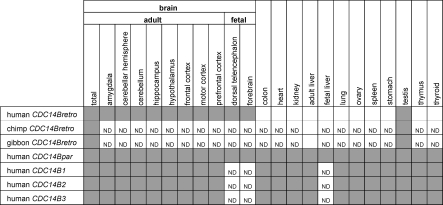
Spatial Expression Patterns of the *CDC14B* Parental and Retrogene Variants Shaded areas indicate expression in a given tissue. “ND” indicates that expression was not determined. Note that weak amplification products of human *CDC14Bretro* were obtained for the following tissues in some of the cDNA panels tested: kidney, lung, ovary, thymus, and thyroid. This suggests that it may be expressed at low levels in these (and perhaps other) tissues in some individuals.

Spatial expression analyses revealed that these variants are widely expressed, as reverse-transcriptase (RT)-PCR products specific to *CDC14Bpar*, *B1*, *B2*, and *B3* were found in all of the 12 tissues tested (Figure 2). In contrast, *CDC14Bretro* is specifically expressed in the adult brain (in all eight regions tested) and testes (Figure 2). In addition, *CDC14Bretro* transcripts were also detected specifically in the human embryonic forebrain, including the dorsal telencephalon, which contains the primordium of the cerebral cortex (Figure 2). Thus, *CDC14Bretro* likely evolved a specific function in the embryonic/adult brain and/or testis since the duplication event in the hominoid ancestor.

To date the origin of its brain-/testis-specific expression, we performed RT-PCR experiments using brain, testis, and liver RNA preparations from chimpanzees and gibbons. Like in humans, we detected *CDC14Bretro* expression in brain and testis from these two apes but not in liver (Figure 2). This suggests that brain- and testis-specific expression was acquired soon after the duplication event in the common hominoid ancestor, although additional ape tissues need be analyzed in the future to confirm this hypothesis.

### Positive Selection of *CDC14Bretro* in the African Ape Ancestor

**Figure 3:**
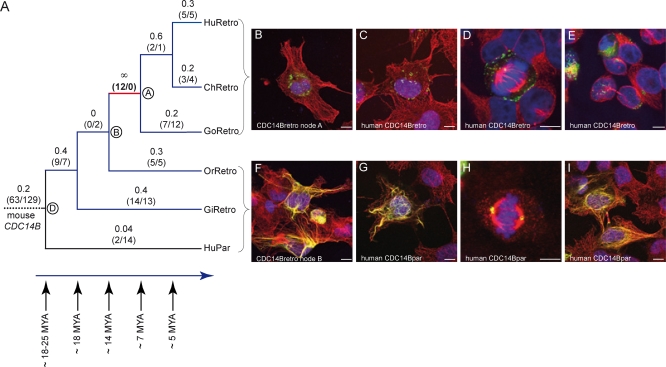
Coding Sequence Evolution of *CDC14Bretro*/*CDC14Bpar* and Subcellular Localization of Encoded Proteins (A) Phylogenetic tree based on the *CDC14B* coding sequences (mouse *CDC14Bpar* sequence is used as an outgroup, see Materials and Methods for details). Approximate divergence times in millions of years (Mya) are shown (estimates based on [15]). Maximum likelihood *d*_N_/*d*_S_ values and the estimated number of nonsynonymous over synonymous substitutions (in parentheses) for each branch are indicated (estimated numbers of substitutions are rounded to the nearest integer). Internal nodes: Duplication event (node D), Before positive selection (node B, sequence from last common great ape ancestor), and After positive selection (node A, last common African ape ancestor). The branch showing positive selection is highlighted in red. Abbreviations: human *CDC14Bretro*, HuRetro; chimpanzee *CDC14Bretro*, ChRetro; gorilla *CDC14Bretro*, GoRetro; orangutan *CDC14Bretro*, OrRetro; gibbon *CDC14Bretro*, GiRetro; human *CDC14Bpar*, HuPar. (B) CDC14Bretro from node A in interphase. COS7 cells transfected with a *CDC14Bretro*-GFP fusion construct (i.e., CDC14B signals in green) are shown. Microtubules are stained in red (anti-beta tubulin antibody). Nuclear DNA was stained with DAPI (blue). (C) Human CDC14Bretro in interphase. (D) Human CDC14Bretro during mitosis. (E) Human CDC14Bretro during cytokinesis. (F) CDC14Bretro from node B in interphase. (G) Human CDC14Bpar in interphase. (H) CDC14Bpar during mitosis. (I) CDC14Bpar during cytokinesis. Only merged images are shown; unmerged images are shown in Figure S3. Similar results were obtained when using either human HeLa or LN229 cell lines (Figure S4). Scale bars = 10 μm.

**Figure 4:**
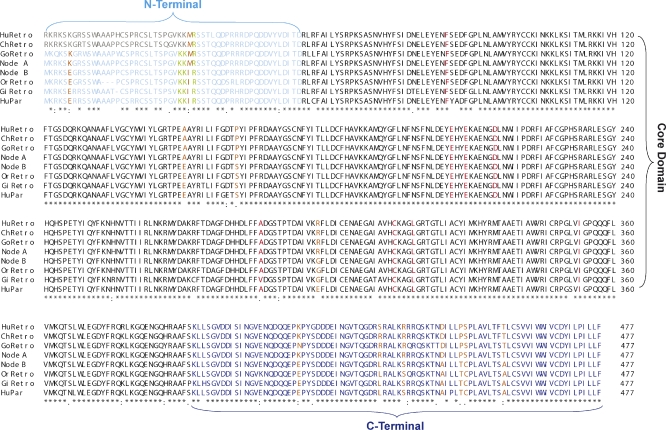
Alignment of Extant and Reconstructed CDC14Bretro and CDC14Bpar sequences N and C termini are highlighted in light and dark blue, respectively. The 12 sites where amino acid changes arose in the common African ape ancestor are highlighted in orange. The amino acid motif KKIR, previously described to be crucial for nuclear localization of the parental variants CDC14B1 and CDC14B2, is highlighted in green. Sites crucial for the phosphatase function of CDC14B [17,35] are highlighted in dark red. The sequence N terminal of the new initial methionine in humans and chimpanzees is marked in gray, as this part of the sequence is likely not translated in these species. The same abbreviations as in the legend of Figure 3 were used to designate CDC14B sequences from the different species.

To trace the functional evolution of *CDC14Bretro*, we first analyzed its coding sequence evolution in a phylogenetic framework. The evolution of *CDC14Bretro* appears to have been shaped mainly by purifying selection, as indicated by the generally low nonsynonymous (*d*_N_) to synonymous (*d*_S_) substitution rate (*d*_N_/*d*_S_; Figure 3A). In sharp contrast, the internal branch leading to the last common ancestor of African apes (human, chimpanzee, and gorilla) reveals a strikingly different pattern, with 12 nonsynonymous and 0 synonymous substitutions, which is indicative of accelerated protein evolution driven by positive Darwinian selection [21]. A conservative maximum likelihood analysis ([22]; see Materials and Methods for details) confirms that the excess of nonsynonymous relative to synonymous substitutions is statistically significant (*p* < 0.05). Furthermore, we found that the nonsynonymous substitutions are nonrandomly distributed in the sequence. Nine of the 12 amino acid changes occurred in the N- or C- terminal part of the protein, whereas the remaining three substitutions occurred in the phosphatase core domain (Figure 4)—a statistically significant difference (Fisher's exact test, two-tailed *p* < 0.01, see Materials and Methods for details).

Interestingly, the nonsynonymous substitutions that occurred in the 5′ end of *CDC14Bretro* during the period of positive selection in the African ape ancestor entailed the origin of a new methionine start codon that allowed the original start codon to be lost in humans and chimpanzees (Figure 4). Analysis of the full-length transcript revealed no other possible in-frame ATG start codon, suggesting that the new downstream start codon is indeed used for initiation of translation. This new methionine start codon disrupts the KKIR motif (replacing the isoleucine residue by a methionine), which was reported to be crucial for the nuclear localization of CDC14B1 [17]. Thus, we hypothesized that the N-terminal substitutions that occurred during the phase of positive selection were associated with a change in subcellular localization of CDC14Bretro in the African ape ancestor. To obtain the necessary background information for testing this hypothesis, we first sought to assess the subcellular localization of CDC14Bpar relative to the other 3' splice variants of the *CDC14B* parental gene, because it represents a novel protein variant with a previously uncharacterized subcellular localization.

### Subcellular Localizations of the Splice Isoforms of the *CDC14B* Parental Gene

Protein localization experiments of the CDC14B splice isoforms with fluorescent reporters fused to their C termini (Figure 1A) show that whereas CDC14B1 (nucleoli) and CDC14B2 (nuclear speckles) localize to the nucleus in interphase cells as previously described [17] (Figure 1B and 1C and Figure S1), CDC14Bpar and the other previously uncharacterized variant CDC14B3 show highly structured filaments throughout the cell, reminiscent of microtubules (MT) (Figures 1E, 1D, and S1). Microtubular staining of transfected cells revealed that these two CDC14B isoforms indeed co-localize with MTs (Figure 1D and 1E and Figure S1). We note that CDC14B1 shows microtubular association in a small proportion of cells, consistent with a previous report that found microtubular co-localization of this variant in 14% of transfected cells [17]. This study also showed that a CDC14B1 mutant protein with an altered KKIR motif shows increased microtubular association.

Together, these results suggest that all CDC14B splice variants have the inherent capacity to co-localize with microtubules, and that in addition to the N terminus, the C-terminal part of CDC14B is crucial for the subcellular localization of the different splice isoforms. In the case of CDC14Bpar and CDC14B3, their specific C-terminal sequences apparently override the nuclear localization signal. This notion is supported by a CDC14Bpar recombinant protein that lacks the C-terminal domain; this truncation mutant localizes to the nucleus (Figure S2).

Thus, we reasoned that the seven amino acid substitutions that occurred during the phase of positive selection in the C terminus of CDC14Bretro (Figure 4) might—in addition to the substitutions in the N terminus (see above)—also have contributed to a potential subcellular localization shift in African apes. Prompted by these observations, we proceeded to directly test the hypothesis of a subcellular localization shift of CDC14Bretro in the African ape ancestor.

### Subcellular Relocalization of CDC14Bretro Due to Positive Selection in the African Ape Ancestor

To this end, we reconstructed the ancestral sequences of the last common ancestor of great apes and African apes, representing the *CDC14Bretro* variants before (node B) and after (node A) the period of positive selection (Figures 3A and 4; see Materials and Methods for details). Subcellular localization experiments of the proteins expressed from these resurrected genes showed that CDC14Bretro from node B co-localizes with MTs during interphase, similar to the parental protein CDC14Bpar (Figure 3F and Figures S3 and S4). In sharp contrast, CDC14Bretro from node A did not localize to microtubules, but rather displayed a new localization pattern with nonfilamentous fluorescent signals around the nucleus (Figure 3B and Figures S3 and S4).

CDC14Bretro from human, chimpanzee, and gorilla shows localization patterns similar to that of the node A sequence (Figure 3B and 3C and Figures S3, S5A, and S5B), whereas CDC14Bretro from orangutans and gibbons shows microtubular association (Figure S5C and S5D), like CDC14Bretro from node B. Thus, except for some small differences between individual extant CDC14Bretro variants, the localization patterns before and after the phase of positive selection have been largely preserved in the Asian and African apes, respectively. This is consistent with the evidence for purifying selection in these lineages (*d*_N_/*d*_S_ < 1, *p* < 10^−2^ for both African and Asian apes; Figure 3A). The evidence of functional preservation by purifying selection further supports functionality of *CDC14Bretro*, in addition to our original evidence of the conservation of its open reading frame by purifying selection (the paucity of non-neutral insertion/deletions; [6]). We note, however, that the evolution of *CDC14Bretro* in the descendant ape lineages has generally been less constrained than that of its parent (Figure S6, *p* < 10^−2^), which may be indicative of (further) functional adaptation and/or some relaxation of selection at certain sites of the CDC14Bretro protein in apes. Although *CDC14Bretro* appears to have accumulated particularly many amino acid substitutions on the gibbon lineage (Figures 3A and 4), the synonymous substitution rate was high on this lineage as well (as also reflected by the low *d*_N_/*d*_S_ ratio). This suggests that CDC14Bretro function was generally selectively preserved by purifying selection, consistent with the preservation of the ancestral MT localization (see above, Figure S5).

Using human CDC14Bretro and CDC14Bpar as representatives of the new and ancestral protein variants, respectively, we show that the subcellular shift is present throughout the cell cycle; whereas CDC14Bpar (as well as CDC14Bretro of the Asian apes; unpublished data) strikingly reorganizes from the microtubules in interphase to the spindle pole during mitosis (Figure 3H and Figure S3) and intercellular bridge during cytokinesis (Figure 3I and Figure S3), human CDC14Bretro lacks association with microtubules also during these phases of the cell cycle (Figure 3D and 3E and Figure S3).

### CDC14Bretro from African Apes Is Associated with the Endoplasmic Reticulum

**Figure 5:**
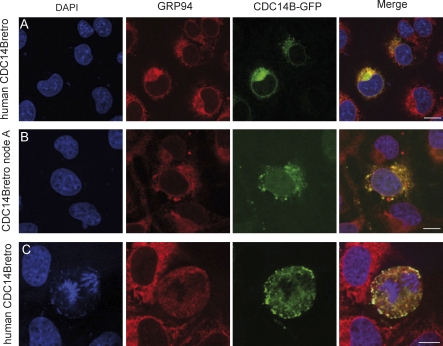
Subcellular Localization of CDC14Bretro from Humans and Node A (A) Human CDC14Bretro in interphase. COS7 cells transfected with two different *CDC14Bretro*-GFP fusion constructs (green) and labeled with an anti-GRP94 antibody (red, a marker of the endoplasmic reticulum) and with DAPI (blue). (B) CDC14Bretro from node A (see Figure 3) in interphase. (C) Human CDC14Bretro during mitosis. Similar results were obtained when using either human HeLa or LN229 cell lines (Figure S8). We note that in addition to the ER, CDC14Bretro from African apes localizes to vesicles that show no co-staining with any of the cellular markers tested (see Materials and Methods) more than 24 h after transfection. Scale bars = 10 μm.

To characterize the new cellular localization of CDC14Bretro from African apes, we co-stained cells transfected with the human *CDC14Bretro* construct using 13 different cellular markers (Materials and Methods and Figure S7). Human CDC14Bretro co-localizes with a marker of the endoplasmic reticulum (ER) in interphase cells (Figure 5A and Figure S8). CDC14Bretro from node A shows a similar ER co-localization pattern (Figure 5B and Figure S8). Furthermore, human CDC14Bretro retains its ER localization during mitosis (Figure 5C), which suggests that CDC14Bretro from humans/African apes does not associate with microtubules at any phase of the cell cycle but remains associated with the ER.

Proteins that are targeted to enter the lumen of the ER have well-described signal peptides with characteristic properties. These include a stretch of 5–15 hydrophobic amino acids that is preceded by a positively charged (basic) amino acid, which allow the protein to pass through the hydrophobic ER membrane. Visual inspection and in silico prediction programs of the N-terminal sequences of CDC14Bretro from African apes reveal no evidence of such signal peptides that could target the protein to the ER or secretory pathway (see Materials and Methods for details). This suggests that CDC14Bretro from African apes localizes to the cytosolic face of the ER, thus probably binding to substrates and/or protein interaction partners on the ER membrane. We conclude that CDC14Bretro evolved a dramatically altered subcellular localization during the cell cycle in the African ape ancestor ∼7–12 Mya [15], shifting from the ancestral microtubular association to a new localization on the cytosolic face of the ER—a process likely driven by positive selection.

### Substitutions Responsible for the Subcellular Shift of CDC14Bretro in African Apes

**Figure 6:**
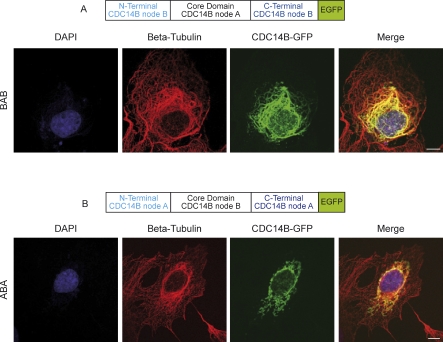
Subcellular Localization of Hybrid Constructs BAB and ABA COS7 cells transfected with hybrid fusion construct BAB and ABA (i.e., CDC14B signals in green). Microtubules stained in red (anti-beta tubulin antibody). Nuclear DNA was stained with DAPI (blue). Scale bars = 10 μm.

To elucidate which substitutions that occurred during the period of positive selection were responsible for the subcellular shift in the African ape ancestor, we generated hybrid constructs of the ancestral *CDC14Bretro* gene variants. Hybrid proteins, where the N and C termini correspond to the node B sequence but the phosphatase core domain corresponds to that of node A (hybrid BAB), show microtubular localization very similar to that seen for the node B protein in ∼53% of cells (Figure 6). A hybrid protein where the N and C termini correspond to the node A sequence and the core domain to that of node B (ABA) loses the filamentous microtubular association in the majority (∼80%) of cells, although some association with MTs is retained (Figure 6). Thus, positively selected substitutions in the N- and C-terminal parts of CDC14Bretro have contributed to the shift in subcellular localization in the African ape ancestor, consistent with our original hypothesis (which was based on the excess of amino acid replacements in the termini). In addition, substitutions in the core domain also affected the capacity for microtubular localization.

Consequently, these results confirm that CDC14Bretro did not “simply” gain an N-terminal signal peptide that could target it to the ER lumen (see above), but was rather shaped by substitutions throughout the protein that lead to a localization on the cytosolic face of the ER. The finding that substitutions in the phosphatase domain of CDC14Bretro from African apes have contributed to the subcellular shift may suggest that the change in subcellular localization was associated with the acquisition of a new function. We note that the subcellular localization of many enzymes depends on their catalytic activity/substrate binding (e.g., [23,24]). Thus, we hypothesize that the subcellular relocalization of CDC14Bretro in the African ape ancestor was due to a change in substrate and/or protein interaction partners.

### Microtubular Stabilization Capacities of Extant and Ancestral CDC14Bretro and CDC14B Variants

**Figure 7:**
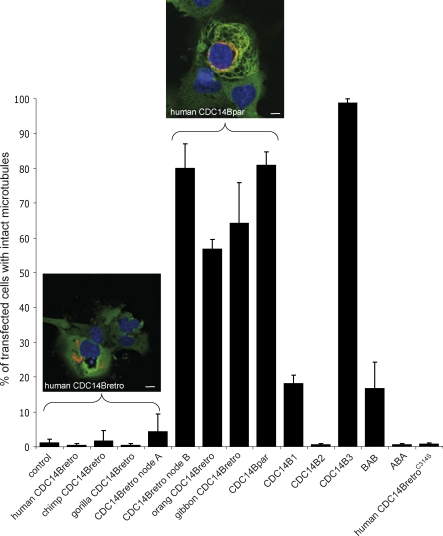
Microtubule Stabilization Capacity of CDC14Bretro and Parental CDC14B Variants Cells transfected with CDC14Bretro/CDC14B were treated with the depolymerizing agent nocodazole (see Materials and Methods for details). All experiments were repeated five times. Representative images of COS7 cells treated with nocodazole 24 h after transfection with human CDC14Bretro and human CDC14Bpar (fused to DsRed), in which microtubules (labeled in green) are stabilized (right image, yellow/green filaments; yellow indicates CDC14Bpar-microtubule co-localization) or degraded (left image) are shown. Statistical analysis reveals significant stabilization differences between the different CDC14B proteins (one-way ANOVA, *p* < 10^−4^). Relevant pairwise comparisons of proteins that show significant stabilization differences (*p* < 0.01, Tukey's Post Hoc test): (i) CDC14Bretro from humans, chimpanzee, gorilla, node A versus CDC14Bretro from orangutan, gibbon, node B, CDC14Bpar, and CDC14B3; (ii) CDC14B1 and CDC14B2 versus CDC14Bpar/CDC14B3; (iii) node B versus BAB/ABA hybrids; (iv) BAB versus ABA; (v) CDC14Bretro from orangutan and gibbon versus CDC14Bretro from node B and CDC14Bpar. Scale bars = 10 μm.

A recent report revealed evidence for some microtubular bundling and stabilizing activity of CDC14B1 in a fraction of cells [17]. Thus, to gain further insights into the functional shift associated with the change of subcellular localization in African apes, we tested the ability of the parental and retrogene variants of CDC14B to stabilize MTs by treating transfected cells with the MT depolymerization agent nocodazole. These analyses show that while CDC14Bpar and CDC14B3 effectively stabilize MTs, the other two, predominantly nuclear parental isoforms (CDC14B1 and 2) show little or no stabilization capacity (Figure 7).

The resurrected node B protein as well as CDC14Bretro from orangutan and gibbon show high stabilization capacity; as high (node B protein), or almost as high (orangutan, gibbon), as that of CDC14Bpar. The somewhat lower capacity of the orangutan and gibbon variants may be due to small, lineage-specific affinity changes. In contrast, CDC14Bretro from node A and its extant descendants have completely lost the ability to stabilize MTs, consistent with the absence of detectable microtubular localization and their likely new functional role on the ER.

As predicted from the localization experiments (loss of MT association), the hybrid construct ABA shows complete loss of MT stabilization activity. In spite of its microtubular association in ∼53% of cells (see above), hybrid construct BAB loses MT stabilization capacity in most (∼85%) of the cells (Figure 7). These results suggest that the localization and MT stabilization capacities are, to some extent, decoupled. The termini seem to be crucial for MT localization, while the core domain is necessary for MT stabilization. Thus, there seems to be a complex interplay of the different parts of the protein; both the protein termini and the core domain are needed for effective MT association and stabilization of the ancestral CDC14B variants. Together, the MT localization and stabilization analyses substantiate the view that most or all of the substitutions that occurred throughout the protein during the period of positive selection in the African ape ancestor were likely adaptive and required for the subcellular/functional shift of CDC14Bretro.

### Conclusion

In this study, we set out to characterize the *CDC14B* cell cycle gene and, in particular, the functional evolution of its daughter gene *CDC14Bretro*. We found that *CDC14B*, which has, as yet, not been characterized in detail, encodes four different splice isoforms that differ with respect to their subcellular localization (nucleus or microtubule-associated) and functional properties (microtubular stabilization capacities) as a consequence of their 3′ splicing patterns (Figures 1 and 7). This result has important consequences, as future studies of CDC14B need to take the presence of different isoforms into account and assess their functional roles separately. We have begun here to functionally characterize one of the novel splice variants, *CDC14Bpar*, and we find that it encodes a protein that is associated with MTs throughout the cell cycle. CDC14Bpar reorganizes from MTs in interphase to the spindle pole during mitosis and possesses a strong microtubular stabilization capacity (Figures 1E, 3G–3I, 7, and Figure S3). Thus, the CDC14Bpar isoform may be involved in mitotic microtubule dynamics and organization.

*CDC14Bpar* spawned a new retroduplicate gene copy, *CDC14Bretro*, which was fixed in the common hominoid ancestor (Figure 1A). It adopted or evolved brain and testis expression soon after the duplication event. A shift toward an enlarged neocortex and enhanced cognitive capacities relative to other primates occurred in the hominoid ancestor, presumably driven by selection for increased social complexity [25]. Conceivably, the fixation of *CDC14Bretro*—like that of another hominoid-specific retrogene [8]—might have been associated with the evolution of the more complex ape brain. In this context, it is interesting that we detected *CDC14Bretro* expression in the early human embryonic brain, which might point to a role of this gene in neural development.

But what functional role might it have obtained in the hominoid brain and/or testis? Our functional analyses—demonstrating conservation of ancestral MT localization and stabilization patterns of CDC14Bretro from the great ape ancestor and the extant Asian apes—suggest that until the evolutionary separation of African and Asian apes ∼12 Mya [15], CDC14Bretro may have been functionally preserved (and advantageous) to provide increased dosage of the CDC14Bpar parental isoform during the cell cycle of these tissues. In the African ape ancestor, a radical shift toward a new localization on the ER then occurred. The subcellular/functional shift of CDC14Bretro was perhaps facilitated by the evolution of increased brain (testis) expression of CDC14Bpar, which might have released selective pressure to retain a second *CDC14B* gene copy with similar functional properties in the African ape ancestor. Although CDC14Bretro apparently retained its phosphatase function (all sites required for its phosphatase activity have remained unchanged), it very likely changed its substrate and/or protein interaction partners after relocalizing to the ER.

The new function of CDC14Bretro from African apes on the ER may be unrelated to the cell cycle, although we note that a cell cycle–related function of CDC14Bretro might not be so unlikely, in view of a growing body of work revealing important ties of the ER and the cell cycle (e.g., [26]). However, the precise new role of CDC14Bretro on the ER of the African ape brain and testis warrants further experimental scrutiny. In any event, the signature of positive selection driving this subcellular/functional adaptation suggests that it is a function that has been beneficial to African apes.

More generally, our study reveals a new mode with respect to how new gene copies may obtain new functional roles. A recent study indicated that new genes that emerge through a complex combination of segmental duplications and genomic rearrangements (including exon gain/loss) may evolve new localization patterns [27]. Our work here reveals that subcellular shifts of new duplicate proteins may occur through rapid and adaptive protein evolution, driven by intense positive selection. But how frequently has the process of subcellular adaptation occurred during evolution?

It has been shown in various individual molecular studies that proteins from the same family may show different subcellular localization patterns in mammals (e.g., [28]). Indeed, a recent global survey (using yeast as a model system) suggests that protein subcellular adaptation represents a rather common mechanism through which duplicate genes may functionally diversify [29]. Thus, we believe that in addition to changes in gene expression and/or biochemical function [30], rapid and selectively driven adaptation to new subcellular compartments—as exemplified by the new hominoid CDC14Bretro protein—may represent a widespread mechanism underlying the evolution of new gene function.

## Materials and Methods

### cDNA libraries.

Total RNA preparations from normal adult human brain, colon, heart, kidney, liver, lung, ovary, spleen, stomach, testis, thymus, and thyroid were purchased from AMSBio, and human amygdala, cerebellar hemisphere, cerebellum, hippocampus, hypothalamus, frontal cortex, motor cortex, and prefrontal cortex from Ambion. Total adult brain-, liver-, and testis-RNA from chimpanzee and gibbon, were extracted from post-mortem tissues, kindly provided by S. Pääbo and colleagues, using the Maxwell 16 Total RNA purification kit (Promega). Total RNA from gorilla (EB(JC)) and orangutan (EB185(JC)) cell lines (ECACC repository, Wiltshire, UK) was extracted using a Maxwell 16 Total RNA purification kit (Promega). RNA from human fetal forebrain, dorsal telencephalon (from day 63–67 stages), and liver (from 19 gestational weeks) was extracted using Trizol and a mirVana RNA extraction kit (Ambion), followed by DNAse treatment with the DNA-free kit (Ambion), according to manufacturer's instructions. Reverse transcription of total RNA was done using SuperScript III first-strand system for RT-PCR (Invitrogen) following the instructions of the supplier. Final RNA concentration per reverse-transcription reaction was 1.25 ng/μl.

### Expression pattern of *CDC14Bretro* and *CDC14Bpar*.

The expression patterns of human *CDC14Bretro* and the four *CDC14B* splice variants were determined by PCR using the cDNA libraries described above. Actin expression was monitored as a positive control. PCR reactions were done using JumpStart DNA Polymerase (Sigma-Aldrich) and primer pairs specific for the different *CDC14Bretro* and *CDC14B* parental gene variants (primer sequences available upon request). cDNA concentration per PCR reaction was 8% (v/v). Amplifications were performed in a Mastercycler gradient (Eppendorf) using a standard cycling protocol. PCR reactions were repeated using independent cDNA preparations from different panels (derived from different individuals).

### 5′-RACE.

To map the 5′ end of human *CDC14Bretro*, we used the FirstChoice RACE-Ready cDNA system (Ambion) following the manufacturer's protocol. An aliquot of 0.5 ng of cDNA was used as template for the PCR. We used a primer specific to the *CDC14Bretro* untranslated region (sequence available upon request) and 5′-RACE outer primer (provided in the kit). The PCR reaction product was further amplified using a nested *CDC14Bretro* UTR-specific primer and 5′-RACE inner primer (kit). The resulting PCR product was purified using the GenElute PCR-cleanup system from Sigma, cloned into the pGEM-T easy vector (Promega), and then sequenced.

### Evolutionary and statistical analysis.

The phylogenetic tree of the *CDC14B* coding sequences was reconstructed using a maximum likelihood procedure (DNAML) as implemented in the Phylip package [31]). The topology of the *CDC14B* sequences in the tree reflects the accepted species phylogeny [15]. Other reconstruction methods, such as Bayesian phylogeny reconstruction using the Markov Chain Monte Carlo method (as implemented in the MrBayes program, [32]), yield identical topologies.

*d*_N_/*d*_S_ ratios in the phylogenetic tree were estimated using the codeml free-ratio model as implemented in the PAML4 package (http://abacus.gene.ucl.ac.uk/software/paml.html) [33]. To test if the observed excess of nonsynonymous substitutions in the lineage leading to African apes was a consequence of positive selection, we first compared a one-ratio model (that assumes an equal *d*_N_/*d*_S_ ratio for all the branches in the phylogeny) to a two-ratio model, where an additional *d*_N_/*d*_S_ value is allowed on the African ape lineage. The two models were compared using the likelihood-ratio test [34], and the two-ratio model was found to fit the data significantly better (*p* < 10^−4^) than the one-ratio model. To test whether the *d*_N_/*d*_S_ value of the branch of the common African ape ancestor was significantly different from 1, we compared its likelihood to that of a model where *d*_N_/*d*_S_ on this lineage was fixed to 1. In addition, we tested statistical significance of the excess of amino acid substitutions in the N- and C-terminal sequences compared to the core domain of CDC14Bretro from the African ape ancestor using Fisher's exact test (nine amino acid substitutions at 139 sites of the termini, 3/338 sites in the core domain). To test for purifying selection (*d*_N_/*d*_S_ < 1) on the descendant African ape (since their last common ancestor) and Asian ape lineages, respectively, we compared the likelihood of models where *d*_N_/*d*_S_ was fixed to 1 to models where *d*_N_/*d*_S_ was estimated from the data. To test for *d*_N_/*d*_S_ rate differences between *CDC14Bretro* from African apes (since their last common ancestor)/Asian apes and *CDC14Bpar*, we compared models where the rate was the same for the two groups to models where the rates were allowed to be different (see also Figure S6).

The ancestral sequences for nodes A and B were reconstructed using a maximum likelihood procedure (one-ratio model, M0) as implemented in codeml (the same sequences are obtained using other standard codeml models such as the free-ratio model). Posterior probabilities for all reconstructed codons of these sequences are high (*p* ≥ 0.99). The same sequences are also obtained using other procedures such as maximum parsimony (unpublished data). We note that posterior probabilities for all reconstructed sites throughout the tree are high (*p* ≥ 0.95).

### Signal peptide screen and subcellular localization prediction.

To screen for potential signal peptides in CDC14Bretro from African apes and predict its subcellular localization in silico, we used several different program packages, including: SignalP (version 3.0; http://www.cbs.dtu.dk/services/SignalP/), TargetP (v1.1, http://www.cbs.dtu.dk/services/TargetP/), pTARGET (http://bioapps.rit.albany.edu/pTARGET/), and PREDATOR (http://bioweb.pasteur.fr/seqanal/interfaces/predator.html). Neither of the programs provides any evidence that CDC14Bretro from African apes has an N-terminal signal peptide targeting it to the ER or secretory pathway.

### Recombinant proteins.

Complete coding sequences for the four alternatively spliced *CDC14B* isoforms were obtained by PCR using human testis cDNA. *CDC14Bretro* coding sequences were obtained by PCR (primers sequences available upon request) using the following primate genomic DNA samples from the ECACC repository (Wiltshire, UK): Human “Caucasian,” chimpanzee (Pan troglodytes), gorilla (Gorilla gorilla), and orangutan (Pongo pygmaeus). The gibbon/siamang (Symphalangus syndactylus) sample was kindly provided by C. Roos. The sequences reconstructed for nodes A and B were synthesized and cloned by GenScript. Hybrids ABA and BAB were obtained through directed mutagenesis by introducing substitutions A149E, P161S, and R298G in the node A and E149A, S161P, and G298R in the node B sequence (all primers and restriction enzymes used are available upon request). All inserts were cloned into pEGFP-N1 or pDsRed-N1 (Clontech) vectors using standard procedures (recombinant proteins expressed from these vectors carry the fluorescent reporters at their C termini).

### Cell culture, transfection, and immunofluorescence.

COS7, HeLa, and LN229 cells were cultivated under standard conditions. 24 h before transfection, cells were plated on 18-mm coverslips in 12-well cluster culture vessels. Transient transfection was carried out using the Lipofectamine Plus method (Invitrogen). For immunofluorescence analyses, cells were fixed in 3% formaldehyde for 10 min at room temperature 24 h after transfection. Monoclonal anti-beta tubulin (Invitrogen), polyclonal anti-GRP94 (Abcam), monoclonal anti-calreticulin (BD transduction laboratory), monoclonal anti-GM130, monoclonal anti-caveolin (Abcam), monoclonal anti-vimentine (Sigma), monoclonal anti-vinculine (Sigma), and monoclonal anti-TGN46 (Abcam) were used as primary antibodies for the subcellular localization analyses. Alexa Fluor 594 goat anti-mouse IgG (H+L) (Invitrogen), Anti-Mouse Polyvalent Immunoglobulins-FITC from goat (Sigma), and Alexa Fluor 594 chicken anti-rabbit IgG (H+L) (Invitrogen) were used as secondary antibodies. Organelles were stained as follow: Golgi and membrane with WGA-Alexa Fluor 568 (Invitrogen), mitochondria with MitoTracker Red CMXRos (Invitrogen), lysosomes with LysoTracker Red DND-99, peroxisomes with the SelectFX Alexa Fluor 488 Peroxisome Labeling Kit (Invitrogen), and nuclear DNA with DAPI (Sigma). Cells were analyzed using a Confocal Microscope Zeiss LSM 510 Meta INVERTED by using a 63-fold oil objective. We used LSM for image analysis.

### Nocodazole experiments.

For the nocodazole washout experiments, COS7 cells were treated with nocodazole (Sigma) at a final concentration of 10 μg/ml for 2 h, 22 h after transfection. The drug was removed by warm phosphate-buffered saline (PBS) treatment. Cells were fixed at 2 min after washout in 3% formaldehyde and then stained with an anti-beta tubulin antibody. To perform blind counting of cells with different phenotypes, a coded name was assigned to each lamelle with respect to the plasmid used for the transfection. For each condition, the proportion of cells with intact microtubules was assessed by counting 50–100 cells at 63-fold magnification over ten arbitrarily chosen areas. Each experiment was repeated five times. Differences between treatment groups were evaluated using ANOVA followed by a Post Hoc (Tukey HSD Test), with significance set at *p* < 0.05.

### Ethical approval.

The human fetal material was used according to the guidelines of the three relevant local Ethics Committees (Erasme Academic Hospital, University of Brussels, and Belgian National Fund for Scientific Research) on Research Involving Human Subjects. Written informed consent was given by the parents in each case.

## Supporting Information

Figure S1 Splice Variants of the *CDC14B* Parental Gene and Subcellular Localization of Encoded Proteins in HeLa and LN229 Cells (A–D) HeLa and (E–H) LN229 cells transfected with *CDC14B*-DsRed fusion constructs (i.e., CDC14B signals in red). Microtubules were stained using an anti-beta tubulin antibody (green) and the nucleus using DAPI (blue). Scale bar = 10 μm.
(336 KB PDF)

Figure S2 Subcellular Localization of the Recombinant Human CDC14Bpar Lacking the C-terminal Domain COS7 cells transfected with human *CDC14Bpar*-DsRed fusion construct (red) and labeled with DAPI (blue). Scale bar = 10 μm.
(99 KB PDF)

Figure S3 Subcellular Localization of CDC14Bretro from Apes and CDC14Bpar This features the same analysis as in Figure 3B-I, but including unmerged images.
(304 KB PDF)

Figure S4 Subcellular Localization of Human CDC14Bretro, Human CDC14Bpar, and CDC14Bretro from Node A and CDC14Bretro from Node B in HeLa and LN229 Cells (A and B) HeLa cells and (C and D) LN229 cells transfected with two different *CDC14Bretro*-GFP fusion constructs (green). Microtubules were stained using an anti-beta tubulin antibody (red) and the nucleus using DAPI (blue). Scale bar = 10 μm.
(262 KB PDF)

Figure S5 Subcellular Localization of the Extant CDC14Bretro Proteins from Apes (A–D) COS7 cells transfected with *CDC14Bretro*-GFP fusion constructs (green) and labeled with an anti-beta tubulin antibody (red) and DAPI (blue). Scale bars = 10 μm.
(336 KB PDF)

Figure S6 Coding Sequence Evolution of *CDC14Bretro/CDC14Bpar*
Phylogenetic tree based on *CDC14B* coding sequences, including *CDC14Bpar* from chimpanzee, gorilla, and orangutan. Maximum likelihood *d*_N_/*d*_S_ values and the estimated number of nonsynonymous over synonymous substitutions (in parentheses) for each branch are indicated (estimated numbers of substitutions are rounded to the nearest integer). Nodes are labeled as in Figure 3. *d*_N_/*d*_S_ on the hominoid *CDC14Bpar* branches (marked in green, *d*_N_/*d*_S_ ∼ 0.02) was found to be significantly different (*p* < 10^−2^) from that on the *CDC14Bretro* descendant branches from either African apes since their last common ancestor (in red, *d*_N_/*d*_S_ ∼ 0.25) or Asian apes (in blue, *d*_N_/*d*_S_ ∼ 0.37). See main text for discussion.
(82 KB PDF)

Figure S7 Subcellular Localization of Human CDC14Bretro COS7 cells transfected with *CDC14Bretro*-GFP fusion constructs (green) and labeled with WGA-Alexa Fluor 568 (labeling the Golgi apparatus, in red) and DAPI (blue). Human CDC14Bretro does not co-localize with the Golgi apparatus. There is also no co-localization of human CDC14Bretro with any of the 11 other markers tested (see Materials and Methods). Thus, it only co-localizes with the GRP94 marker used to stain the ER (see main text). Scale bars = 10 μm.
(116 KB PDF)

Figure S8 Subcellular Localization of CDC14Bretro from Humans and Node A in HeLa and LN229 Cells (A and B) HeLa cells and (C and D) LN229 cells transfected with two different *CDC14Bretro*-GFP fusion constructs (green) and labeled with an anti-GRP94 antibody (red, a marker of the endoplasmic reticulum) and with DAPI (blue). Scale bar = 10 μm.
(202 KB PDF)

### Accession Numbers

The GenBank (http://www.ncbi.nlm.nih.gov/Genbank/) accession numbers for genes discussed in this paper are: *CDC14B1* cDNA (AF023158); *CDC14B2* (AF064104); *CDC14Bpar* (EF611343); *CDC14Bretro* (termed *CDC14B2* in GenBank) human (DQ120635), chimpanzee (DQ120636), gorilla (DQ120637), orangutan (DQ120638), and siamang (EF606887).

The Ensembl (http://www.ensembl.org) accession code for *CDC14B3* is ENST00000265659.

## Abbreviations

ER: endoplasmic reticulum
MT: microtubule
Mya: million years ago
