# Supplementary material for: Birth and Rapid Subcellular Adaptation of a Hominoid-Specific CDC14 Protein
Source: PLoS Biol. 2008 Jun 10;6(6):e140. doi: 10.1371/journal.pbio.0060140 (PMC2422853; doi:10.1371/journal.pbio.0060140)
Supplement: Supplementary file 1 [file pbio.0060140.sg001.pdf]

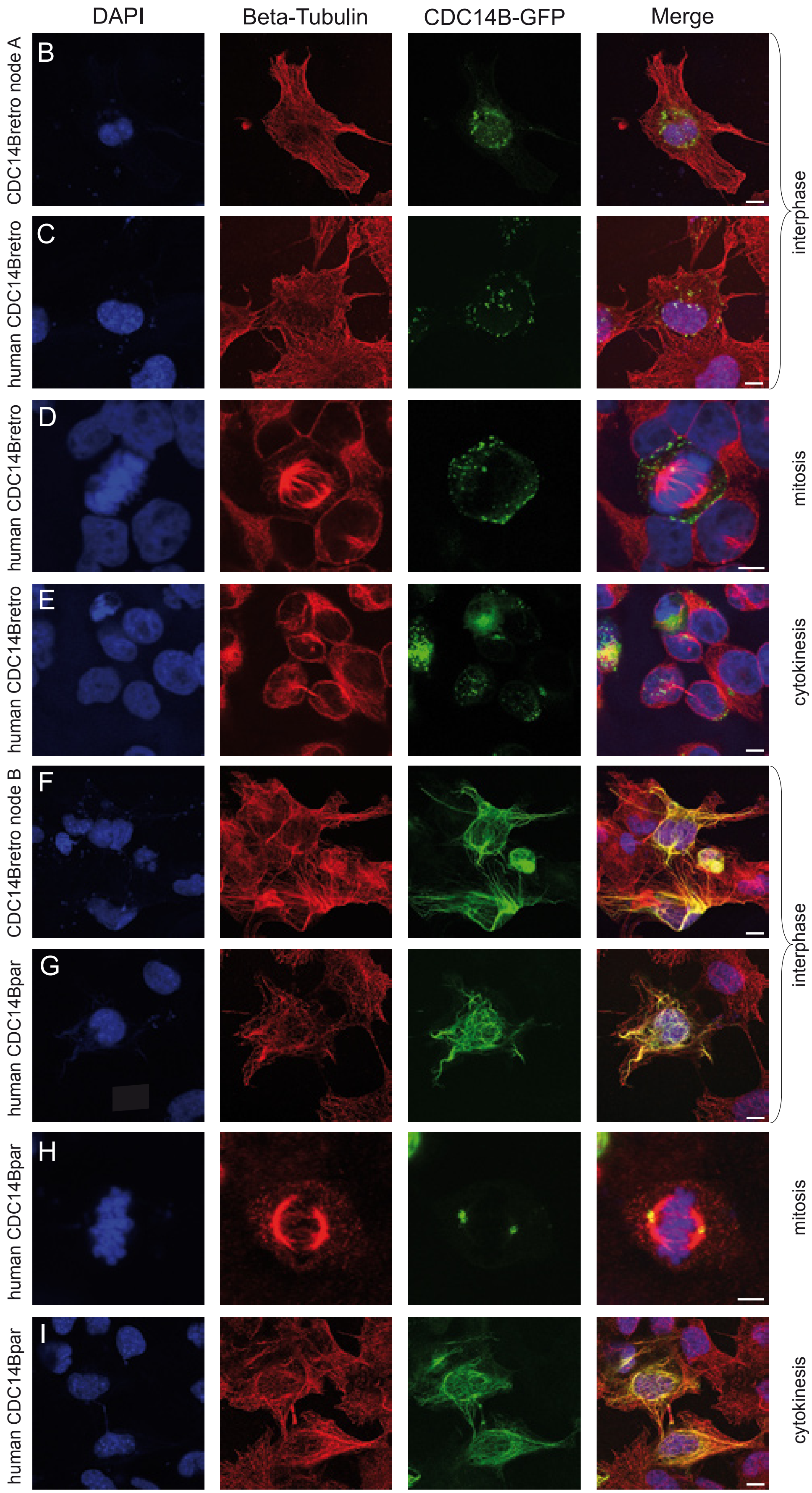
DAPI
Beta-Tubulin
CDC14B-GFP
Merge
CDC14Bretro node A
B
human CDC14Bretro
C
interphase
human CDC14Bretro
D
mitosis
human CDC14Bretro
E
cytokinesis
CDC14Bretro node B
F
human CDC14Bpar
G
interphase
human CDC14Bpar
H
mitosis
human CDC14Bpar
I
cytokinesis
