# Supplementary material for: Birth and Rapid Subcellular Adaptation of a Hominoid-Specific CDC14 Protein
Source: PLoS Biol. 2008 Jun 10;6(6):e140. doi: 10.1371/journal.pbio.0060140 (PMC2422853; doi:10.1371/journal.pbio.0060140)
Supplement: Supplementary file 2 [file pbio.0060140.sg002.pdf]

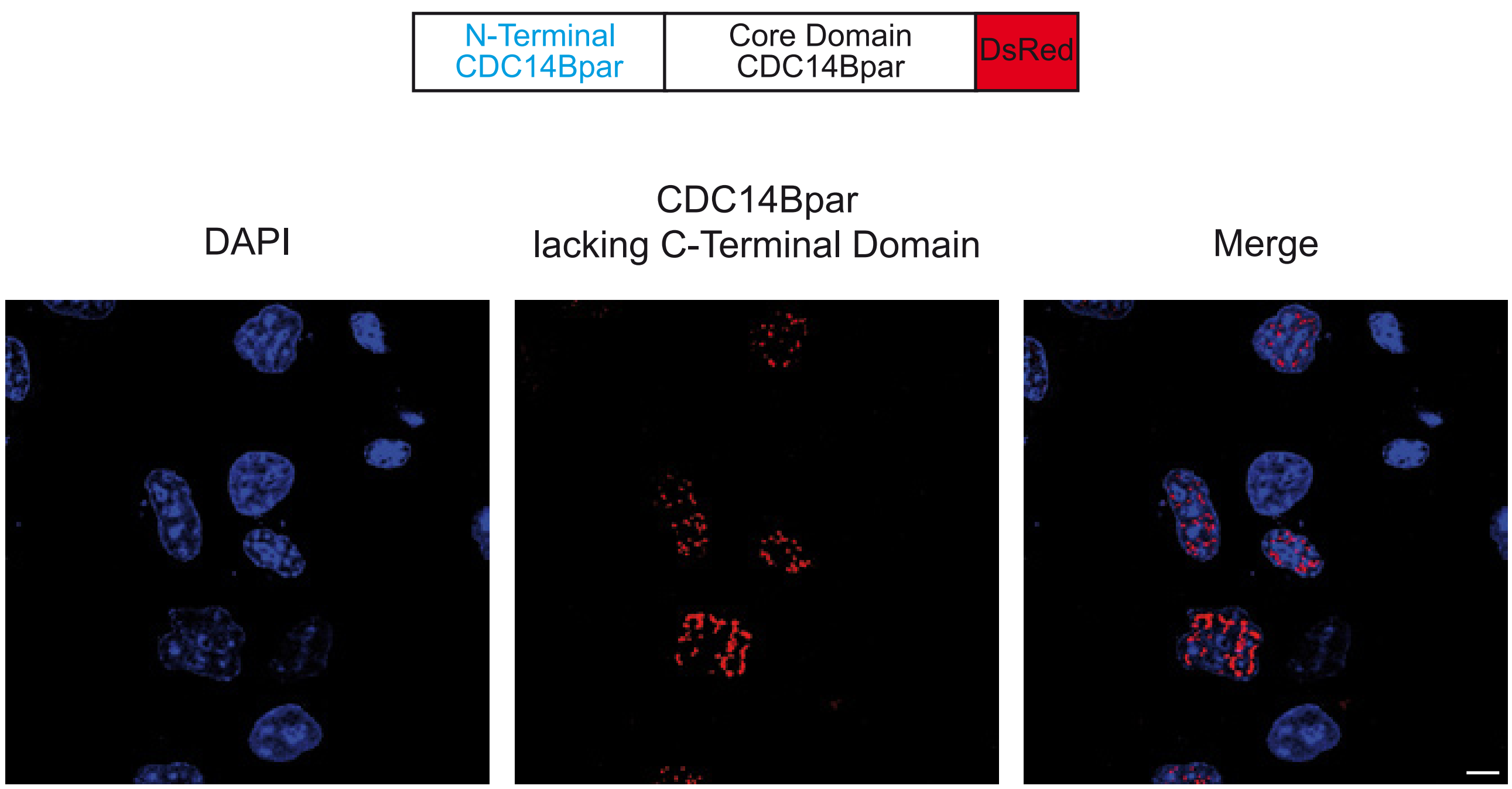
N-Terminal CDC14Bpar
Core Domain CDC14Bpar
DsRed
DAPI
CDC14Bpar lacking C-Terminal Domain
Merge
