# Supplementary material for: Birth and Rapid Subcellular Adaptation of a Hominoid-Specific CDC14 Protein
Source: PLoS Biol. 2008 Jun 10;6(6):e140. doi: 10.1371/journal.pbio.0060140 (PMC2422853; doi:10.1371/journal.pbio.0060140)
Supplement: Supplementary file 3 [file pbio.0060140.sg003.pdf]

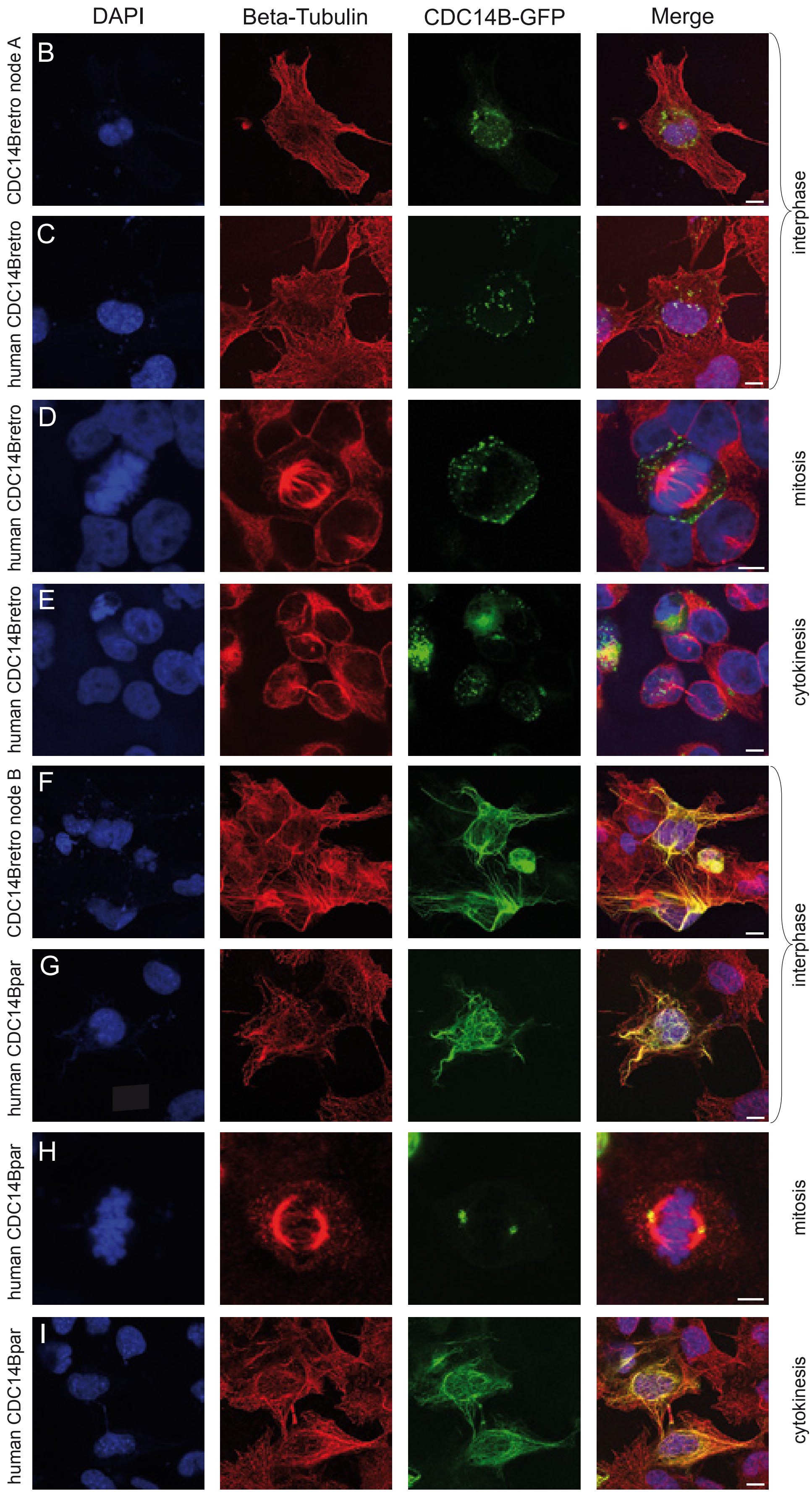
DAPI
Beta-Tubulin
CDC14B-GFP
Merge
CDC14Bretro node A
human CDC14Bretro
human CDC14Bretro
human CDC14Bretro
CDC14Bretro node B
human CDC14Bpar
human CDC14Bpar
human CDC14Bpar
B
C
D
E
F
G
H
I
interphase
mitosis
cytokinesis
interphase
mitosis
cytokinesis
