# Supplementary material for: Birth and Rapid Subcellular Adaptation of a Hominoid-Specific CDC14 Protein
Source: PLoS Biol. 2008 Jun 10;6(6):e140. doi: 10.1371/journal.pbio.0060140 (PMC2422853; doi:10.1371/journal.pbio.0060140)
Supplement: Supplementary file 4 [file pbio.0060140.sg004.pdf]

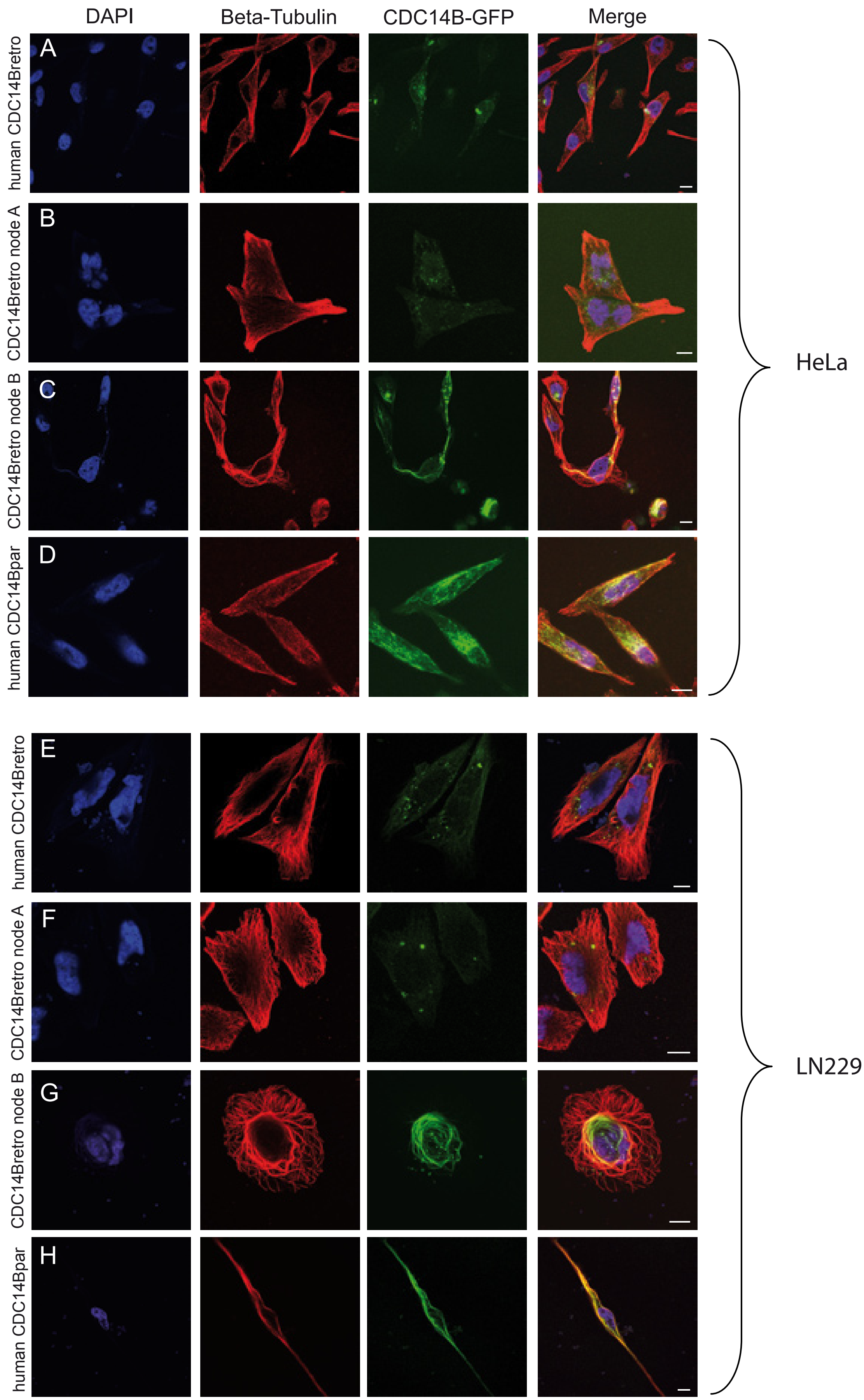
DAPI
Beta-Tubulin
CDC14B-GFP
Merge
A
human CDC14Bretro
B
CDC14Bretro node A
C
CDC14Bretro node B
D
human CDC14Bpar
HeLa
E
human CDC14Bretro
F
CDC14Bretro node A
G
CDC14Bretro node B
H
human CDC14Bpar
LN229
