# Supplementary material for: Birth and Rapid Subcellular Adaptation of a Hominoid-Specific CDC14 Protein
Source: PLoS Biol. 2008 Jun 10;6(6):e140. doi: 10.1371/journal.pbio.0060140 (PMC2422853; doi:10.1371/journal.pbio.0060140)
Supplement: Supplementary file 5 [file pbio.0060140.sg005.pdf]

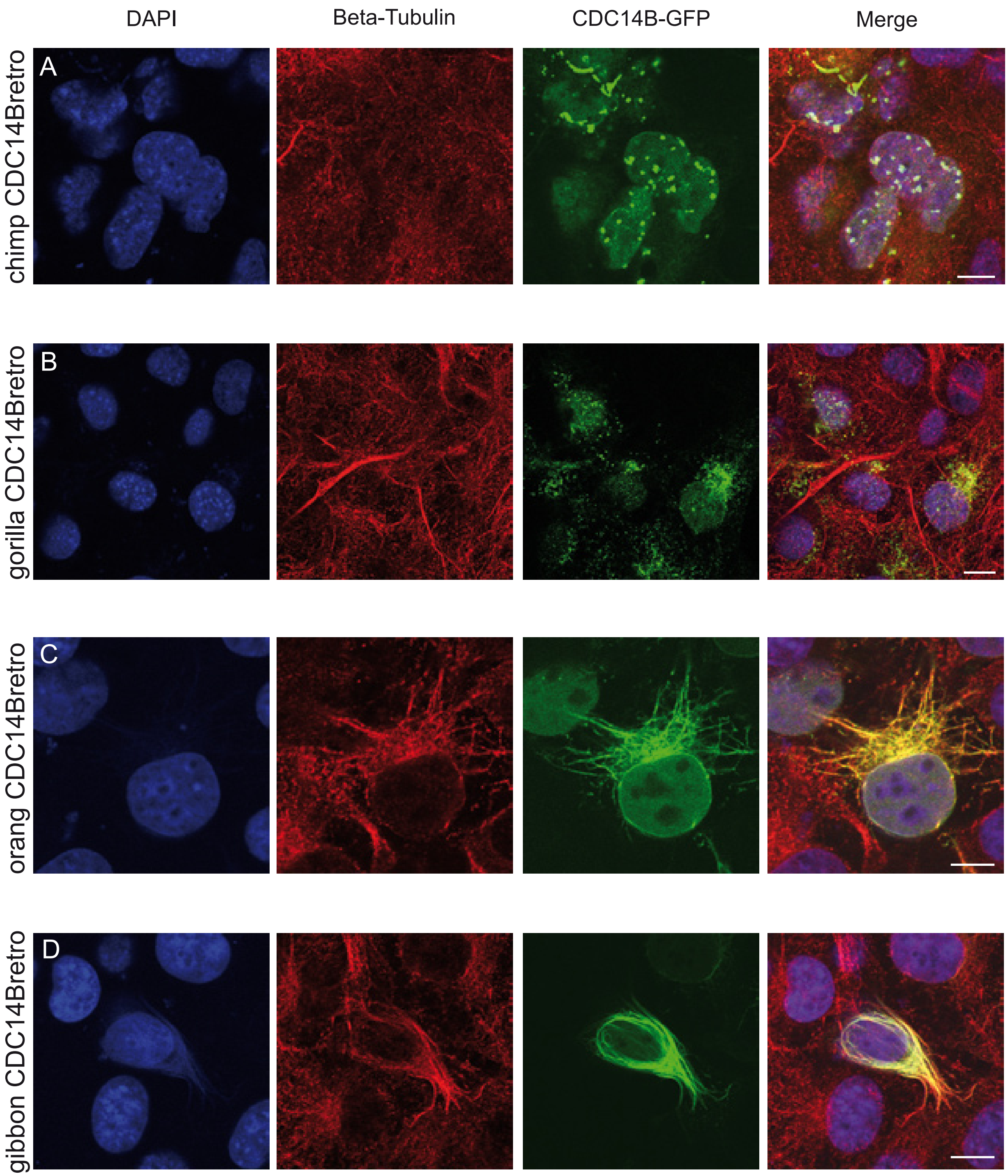
DAPI
Beta-Tubulin
CDC14B-GFP
Merge
chimp CDC14Bretro
gorilla CDC14Bretro
orang CDC14Bretro
gibbon CDC14Bretro
A
B
C
D
