# Supplementary material for: Birth and Rapid Subcellular Adaptation of a Hominoid-Specific CDC14 Protein
Source: PLoS Biol. 2008 Jun 10;6(6):e140. doi: 10.1371/journal.pbio.0060140 (PMC2422853; doi:10.1371/journal.pbio.0060140)
Supplement: Supplementary file 6 [file pbio.0060140.sg006.pdf]

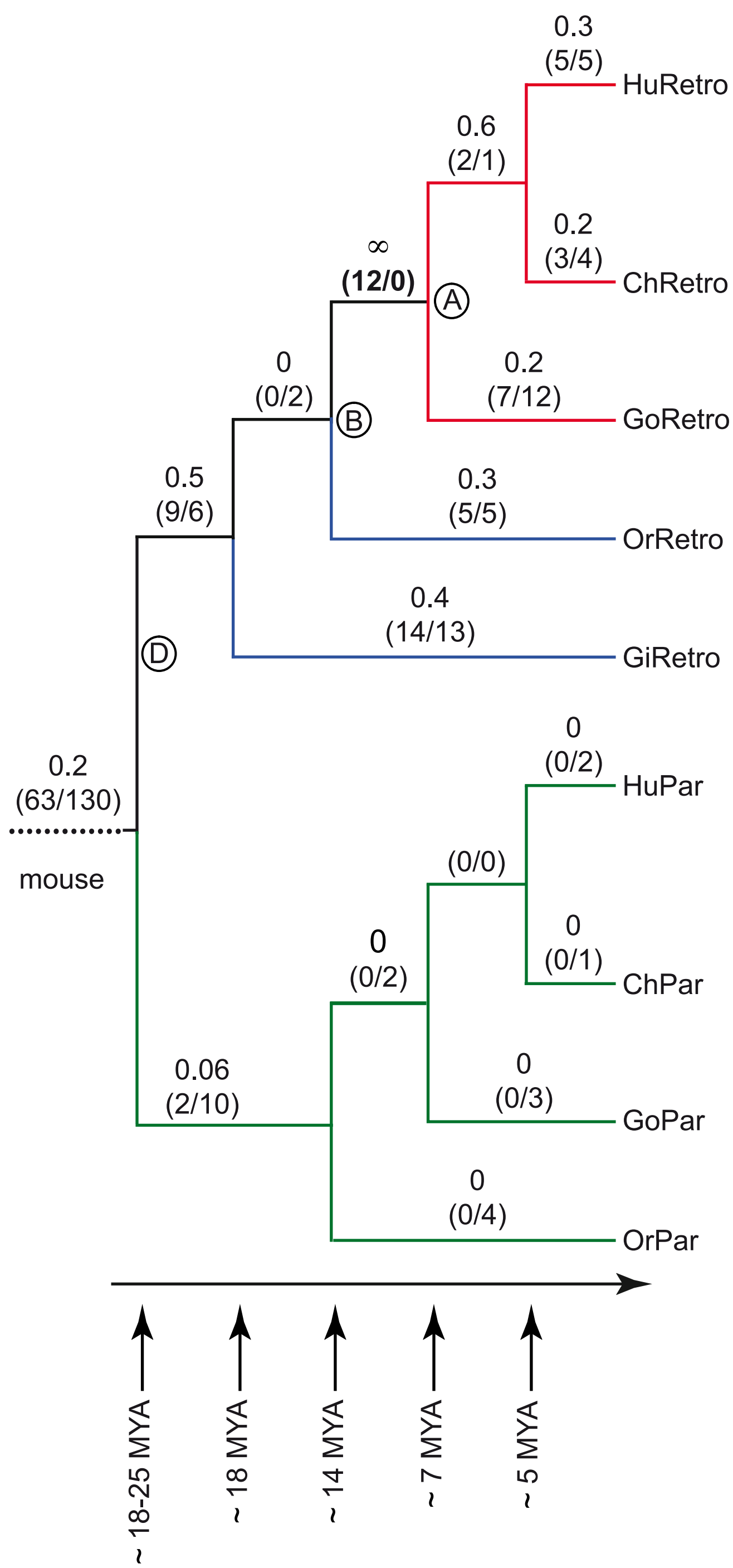
0.3
(5/5)
HuRetro
0.6
(2/1)
0.2
(3/4)
ChRetro
∞
(12/0)
A
0
(0/2)
B
0.2
(7/12)
GoRetro
0.5
(9/6)
0.3
(5/5)
OrRetro
0.4
(14/13)
GiRetro
D
0.2
(63/130)
mouse
0
(0/2)
HuPar
(0/0)
0
(0/1)
ChPar
0
(0/2)
0.06
(2/10)
0
(0/3)
GoPar
0
(0/4)
OrPar
~ 18-25 MYA
~ 18 MYA
~ 14 MYA
~ 7 MYA
~ 5 MYA
