# Supplementary material for: Birth and Rapid Subcellular Adaptation of a Hominoid-Specific CDC14 Protein
Source: PLoS Biol. 2008 Jun 10;6(6):e140. doi: 10.1371/journal.pbio.0060140 (PMC2422853; doi:10.1371/journal.pbio.0060140)
Supplement: Supplementary file 8 [file pbio.0060140.sg008.pdf]

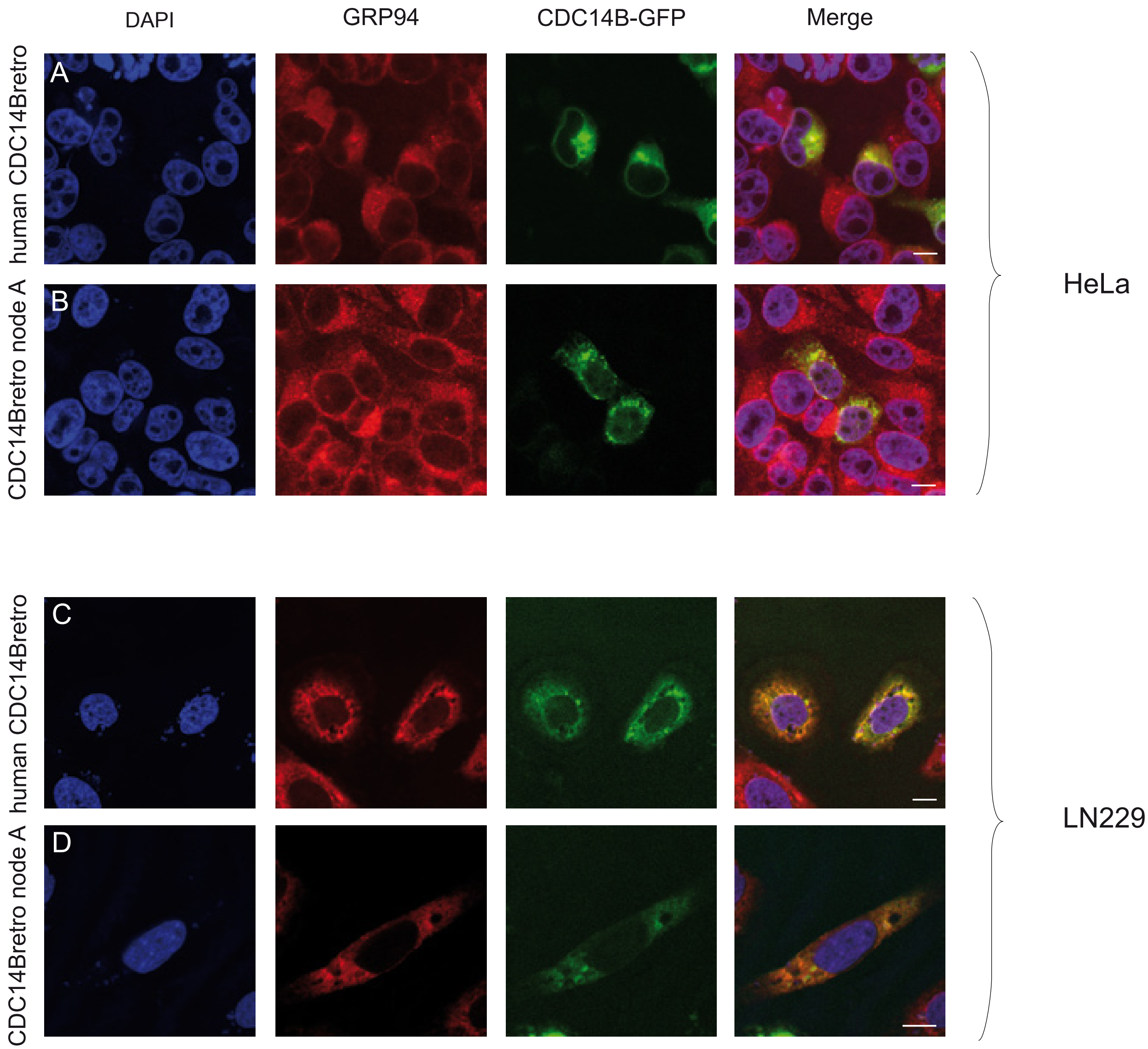
DAPI
GRP94
CDC14B-GFP
Merge
human CDC14Bretro
A
CDC14Bretro node A
B
HeLa
human CDC14Bretro
C
CDC14Bretro node A
D
LN229
